# Functionality versus dimensionality in psychological taxonomies, and a puzzle of emotional valence

**DOI:** 10.1098/rstb.2017.0167

**Published:** 2018-02-26

**Authors:** Irina Trofimova

**Affiliations:** 1 CILab, Department of Psychiatry and Behavioral Neurosciences, McMaster University, 92 Bowman Street, Hamilton, Ontario, Canada L8S 2T6; 2 OISE, Department of Applied Psychology & Human Development, University of Toronto, Toronto, Ontario, Canada

**Keywords:** neurotransmitters, temperament, functional constructivism, Positive/Negative Affects, opioid receptors, FET model

## Abstract

This paper applies evolutionary and functional constructivism approaches to the discussion of psychological taxonomies, as implemented in the neurochemical model Functional Ensemble of Temperament (FET). FET asserts that neurochemical systems developed in evolution to regulate functional-dynamical aspects of construction of actions: orientation, selection (integration), energetic maintenance, and management of automatic behavioural elements. As an example, the paper reviews the neurochemical mechanisms of interlocking between emotional dispositions and performance capacities. Research shows that there are no specific neurophysiological systems of positive or negative affect, and that emotional valence is rather an integrative product of many brain systems during estimations of needs and the capacities required to satisfy these needs. The interlocking between emotional valence and functional aspects of performance appears to be only partial since all monoamine and opioid receptor systems play important roles in non-emotional aspects of behaviour, in addition to emotionality. This suggests that the Positive/Negative Affect framework for DSM/ICD classifications of mental disorders oversimplifies the structure of non-emotionality symptoms of these disorders. Contingent dynamical relationships between neurochemical systems cannot be represented by linear statistical models searching for independent dimensions (such as factor analysis); nevertheless, these relationships should be reflected in psychological and psychiatric taxonomies.

This article is part of the theme issue ‘Diverse perspectives on diversity: multi-disciplinary approaches to taxonomies of individual differences’.

## 1. Functional constructivism in psychological and biological sciences

### (a) Components versus dimensions in psychological taxonomies

This paper compares two approaches to psychological taxonomies: functional (componential) and dimensional; it also reviews how these two approaches deal with the problem of interlocking between the main dimensions of these taxonomies—energetic and emotionality.

It might sound unscientific and almost anecdotal that at the beginning of the 3rd millennium we have to consult the writings of two doctors, Hippocrates and Galen, who deserve credit for at least three insights we find useful in our current discussions of psychological taxonomies. They achieved this 2500 years ago in spite of having no access to modern technology such as brain scanning, neurochemistry or even basic blood analysis, and with strong prohibitions on anatomic dissections. These insights are:

(1) *Componential approach*: their theory suggests that individual differences are a mixture (in Latin—‘temperamentum’) of several interacting fluid components, with the ratio between them determining the specific types. A balanced mixture of these vital chemical components creates normality, whereas imbalanced ‘temperamentum’ causes at least four identifiable patterns of behaviour: choleric (aggressive and/or impulsive), melancholic (depressed), phlegmatic (socially detached and/or withdrawn) and sanguine (overconfident, cheerful, social).
(2) *Described typology*: from the time of Hippocrates and Galen, scholars and clinicians have agreed that impulsivity/aggression, depression, social detachment, high sociability or mania exhibits a peculiar consistency once identified in someone's behaviour, suggestive of the presence of underlying biological factors. Extreme expressions of these four ‘temperamentum’ are seen in mental disorders and are identified in the Diagnostic and Statistical Manual of Mental Disorders (DSM-5) as (respectively) ADHD and Conduct Disorders (codes 314 and 312), Depressive, Conversion or Dysthymic Disorders (296.20-36 and 300), Autism Spectrum or Communication Disorders (299 and 315) and Manic episodes (296.01-06 and 296.41-56) or Histrionic Personality Disorder (310.50). Perhaps these Greek doctors deserve credit for the first condensed version of the DSM/ICD.
(3) *Chemical nature*: the extensive field of psycho-pharmacology is a ‘proof of concept’ of associations between neurochemical systems and mental disorders. The same neurotransmitter (NT) systems studied there have also been linked to many temperament traits (such as impulsivity, sensation seeking, neuroticism, endurance, plasticity, sociability or extraversion, etc.)

Research into the structure of temperament and the development of new DSM/ICD classifications is mutually beneficial since they both work on taxonomies of the most consistent characteristics of behaviour. If individual differences are indeed based on neurotransmitter systems, we must first classify the functional roles of these systems in behavioural regulation, and then use this classification to try to derive the structure of psychological taxonomies. This very idea was proposed by Galen 2500 years ago. He described temperament types as the product of interplay between four chemical systems contributing to human character. Modern neurochemistry has identified somewhat more than four such systems: three monoamine neurotransmitters (MA), acetylcholine (ACh), GABA, glutamate, hormones, endogenous opioids system, 100+ neuropeptides, and other chemical systems such as calcium, brain-derived neurotrophic factor (BDNF), cAMP response element-binding protein (CREB), other proteins, etc. [1]. Moreover, each neurotransmitter has a diversity of receptor types differing in their functionality. For instance, there are five types of dopamine (DA), 11 types of noradrenalin (NA), five types of GABA, eight types of acetylcholine and 14 types of serotonin (5-HT) receptors [1]. These receptors can be either excitatory or inhibitory, depending on: their type and location, the intensity of their stimulation, or the duration of their exposure to agonists and antagonists [1–7]. Galen's idea was prescient but how can we sort out this dynamic, mutually regulating plethora of functional neurochemicals? The first attempts in doing so emerged some decades ago [8–12] and this work is still in progress (for example, [13–19]).

### (b) Functional constructivism

We follow an approach that has been known since Heraclitus and is summarized as *Functional Constructivism* (FC). If the dynamical nature and diversity of NT systems is not sufficiently challenging, the FC approach makes the problem of constructing human taxonomies even worse. In FC, all behaviours are regarded as transient, generative processes which are constructed anew each time based on both the available capacities and the situational demands on the system. This generative principle stands even when behaviour looks repetitive (i.e. follows the same script) because, neurophysiologically speaking, nothing is actually being repeated. The most prominent evidence that specifically demonstrated the constructive principles of behaviour was discovered in experiments in kinesiology conducted by Nikolay Bernstein in the mid-1930s [20]. FC concepts were subsequently used in cybernetics [21], with more evidence appearing in neurophysiology [22–27], neurochemistry [2,17,28–32], developmental and educational psychology [33–36], ecological psychology [37,38], mathematical modelling in psychology [39,40], psychology of perception [41,42], cognition [43–46] and emotions [47–50].

The main FC principles are: (i) all consistent phenomena are *constructed* based on available resources and environmental demands; (ii) they are *transient*; they emerge, change and disappear; (iii) in line with the concept of *contingency cycles* [51], their construction depends on many levels of organization; (iv) they are preceded by *multiple* ‘*drafts*’ that never come to fruition, so *not all of their dynamics can be observed*; (v) drafts, alternative and completed performances of cycles are selected *simultaneously at multiple levels*, creating ‘diagonal’ iterative dynamics [31,32]; (vi) not all constructions achieve a good fit to demands; nevertheless, they are still produced; (vii) owing to contingent and feedback relationships between components of contingency cycles, there is a strong *functional and physical overlap* [31,32]. For the past 30 years, scientists have tried to adapt the formal language of nonlinear dynamics and of open and dissipative systems to biology; however space does not permit us to describe the numerous proposed formalisms [31,32,39–41,52].

What does FC mean for the development of psychological taxonomies? Since we are discussing biological systems of psychological diversity, it makes sense to consult biology and evolutionary theory, where similar FC and ‘emergence’ principles were independently described (for example, [53–55]). One of the useful concepts from these theories that pertains to the nature of psychological traits is ‘contingency cycles’ [51]. Similar to traits, cycles (for example, in ecology) have observable consistency; however, they follow not one but several scenarios with several contingencies. Similar to many Multilevel Selection Theories (for example, [56–58]), including the theory of diagonal evolution [31,32], reports in psychophysiology and psychology describe FC principles of strong functional overlaps between the components of natural systems, constructive and selective natural processes operating simultaneously at several levels of organization [17,18,31–50]. This means strong inter-relatedness between components of highly integrated natural systems. The multi-level integration of psychological systems makes correlations (including factor analysis that is based on correlation matrices) rather useless units of analysis for taxonomies, as their causal links cannot show more than that 'everything depends on everything'. More fruitful in creating taxonomies is the analysis of those principles that underlie its classes, as well as analysis of the main selecting factors reinforcing regulatory neurophysiological systems in evolution. After all, these systems did not evolve overnight, and there are principled reasons why their development went in very specific directions. We believe that the universal features of everyday animal/human activities were such reinforcing factors, and so perhaps the generating architecture (universal functional components) for constructing actions should be used as the main principle of our taxonomies.

### (c) Functional Ensemble of Temperament: a neurochemically based, activity-specific model

Using the functionality approach, a common-sense Grand Plan would be: (a) to identify universal regulatory aspects in the construction of human actions for which (b) links to specific neurophysiological systems have been well-established, (c) to compare them with observations from temperament research and classifications in differential psychology, and (d) to look at the variability of the resulting traits in healthy and mentally ill people.

**Figure 1.**
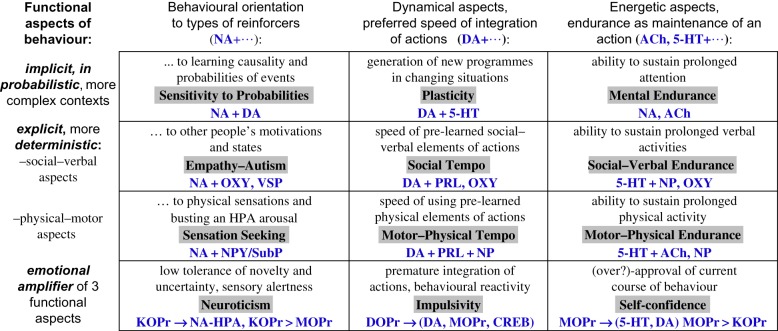
Functional Ensemble of Temperament (FET) model linking traits to teams of NT. Bold shadowed text highlights the names of temperament traits, expression of which depends on a balance within indicated NT systems. 5-HT: serotonin; DA: dopamine; NA: noradrenalin; ACh: acetylcholine; GH: growth hormone; SOM: somatostatin; PRL: prolactin; OXY: oxytocin; SubP: Substance P; NPY: neuropeptide Y; KOPr, MOPr, DOPr: kappa-, mu- and delta-opioid receptors correspondingly. (Online version in colour.)

The call for a ‘functional’ instead of a ‘structural’ approach is not new and came, in fact, soon after scientific psychology was born. In 1905, Robert Yerkes specifically distinguished between structural and functional ‘criteria of psychic’ that nowadays we call dimensions [59, p. 144]. Simonov [60] later suggested that temperament types and traits, especially those related to emotionality, could be derived from probabilistic and motivational aspects of human actions, and linked the regulation of these aspects to four brain regions. A functional approach to the classification of temperament traits was also offered by Rusalov [61] (see also his contribution to this theme issue). Rusalov based his functional model on his electro-physiological studies using Anokhin's [22] model of functional systems (fulfilling goal (a) in our Grand Plan) and his own studies (fulfilling goal (b)). Later, his model was integrated with studies in neurochemistry and with the main models of temperament and differential psychology (fulfilling all goals a–d) [17,18,61–64]. Similar to Rusalov [61], we used the functional architectures of human action that had the most consensus in several behavioural sciences. More specifically, insights from kinesiology were used because this science analyses how humans and animals construct their actions. This line of research began with the classical studies of Bernstein [20] in the 1930s and his original methods of recording motions using mounted sensors. In psychophysiology, Anokhin [22] studied the way behaviour is constructed in cats whose affector- and effector-nerves innervating their muscles (and also their muscles *per se*) had been rearranged. The neuronal and neuropsychological changes were recorded while the cats were learning to use their unusually wired bodies. FC models arising from different disciplines having different architectural compositions converged on the idea that construction cycles of behaviour consist of at least three universal dynamical components: orientation, integration-programming of actions and energetic maintenance of actions (three columns of figure 1).

Congruent with goal (b), several models in functional neuroanatomy and clinical neuropsychology (for example, [20–23,44,45,47,65]), as well as the analysis of functionality of NT systems, identified these three functional aspects of behaviour [17,18].^1^ As discussed in previous publications, NA systems appeared to be regulating aspects of behaviour that relate to orientation and the expansion of degrees of freedom in behaviour; DA appeared to be regulating the assignment of priorities, salience and sequencing to behavioural elements, necessary for behavioural plasticity; 5-HT appeared entangled with the endocrinal regulation of energetic maintenance [28] whereas ACh was associated with the regulation of sustained attention (for reviews see [1–6,8–13,15–19,28,65]). This view on functionality of NA, DA and ACh is widely accepted; however the 5-HT system is rarely praised for its evolutionarily-old role in regulation of many body and brain functions (with exception of [28]). Meanwhile, 5-HT deficiency is often associated with a wide range of dysfunctional behaviour—lack of impulse control [9–11,16,19], aggression, depression, anxiety, social avoidance and Obsessive–Compulsive Disorder (OCD)—so this non-specificity might just be a reflection of low energetic capacities of an individual to construct behaviour adequate to a situation. The higher density of 5-HT1A receptors in the thalamus in people with higher neuroticism [66] might be explained by dysregulation in the NA system (since activation of 5-HT1A receptors (unlike other types of 5-HT receptors) in subcortical systems was found to increase NA [5,6]). This position on the role of 5-HT might be controversial, and the subject of future discussions. The links between the DA system and sensation/novelty seeking (see the Farde *et al*. contribution to this volume [67]) might be explained by test items measuring this trait which often relate to integrative, initiatory aspects of behaviour.

The FC principle of the multi-level overlapping of functional systems found validation when the idea of several ‘levels of control’ was independently described in several behavioural sciences. Behaviour appeared to be regulated by different neuroanatomic systems for well-known (deterministic) and complex/novel (probabilistic) situations [2,3,17,18,20,21,27,36,42–45,61,65,68]. A correlational approach would not be capable of separating these levels of control in behavioural construction since they are tightly interdependent: an increase of situational complexity (and/or novelty) moves the control over the construction of behaviour to neocortical systems whereas a decrease of such complexity/novelty passes this control to the subcortical structures [27,36]. All three MA and also ACh systems demonstrate neuroanatomic branching into limbic, basal ganglia and neocortical levels of brain structures [18], plus differences in functionality of dorsal and ventral striatum [69] appeared to be in line with the idea of such levelling.

Moreover, development of the verbal (fronto-temporal) cortex has often been linked by evolutionists to increased social activities of early humans. The development of mechanisms for optimizing peer interactions within multi-agent systems is considered here to be a universal ‘horizontal’ FC principle of construction of living systems and not just a product of human evolution [31,32]. Several temperament researchers suggested an activity-specific differentiation of traits regulating physical, verbal–social, and mental aspects of the tasks [61–63,70,71]. The functional specificity of cortical areas for verbal processing, abstract thinking and management of physical aspects of behaviour, as well as the role of oxytocin and vasopressin hormones in social-affiliative aspects of behaviour [72], support this activity-specific approach.

Integration of (a)–(c) within the functional constructivism approach resulted in the neurochemical model Functional Ensemble of Temperament (FET) [17,18], initially proposed in 2007 as the STQ-77 structure [62] (figure 1). The goal (c) was achieved through the classification of the traits proposed in the most commonly used models of differential psychology using these three functional aspects as a frame of reference ([17]; see electronic supplementary material, S1). The FET summarizes 12 biologically-based components of behavioural regulation in a 3 × 4 ensemble, in which components regulate each other's performance. None of the components (temperament traits) is proposed to be regulated by a single neurotransmitter system. Instead, each component of the FET is linked to an interplay between specific NT systems, similar to the composition of elementary particles by quarks. These NT systems work in teams, which are specific to each trait, with dominant NTs for each team. In this model, Emotionality is presented as an interplay between three types of opioid receptor (OR) systems acting as amplifiers of the dynamical aspects (i.e. of sensitivity, energetic and dynamic characteristics), discussed in more detail below.

### (d) Role of opioid receptors in emotionality traits

Since this paper reviews the controversy around emotionality-related dimensions of taxonomies, let us examine the neurochemical systems that have been linked to the experience of pleasure, dysphoria and anxiety: OR systems. Endogenous OR systems are part of the G-protein coupled receptor (second messenger) system (GPCR) regulating transmission between many brain NTs, including MA. Dozens of types of endogenous ORs have been found, but the majority of them were classified into three groups: mu-ORs (MOPrs) binding endorphins, kappa-ORs (KOPrs) reacting to dynorphins, and delta-ORs (DOPrs) binding encephalins [7,30,73]. ORs were considered first in the context of their direct effects on mood (pleasurable or analgesic) when they are administered to the body from external sources. Later, as with other NTs, it was found that the body is capable of producing endogenous binding agents (in this case opiates) and of changing the density of endogenous ligands.

The density of receptors has been linked to dispositional emotional states that are independent of the situations eliciting them. The density of ORs can increase (*upregulation*) if they fail to receive the expected amount of binding agents, or decrease, as a result of chronic stimulation by agonists (*downregulation* and desensitization of receptors). A single administration of opiates often causes a temporary imbalance that usually is restored by a chain of recovery mechanisms. Up- or downregulation of receptors is observed mostly after a repetitive/chronic imbalance between the supply of binding agents and the density of the receptor [35,73–76]. When the administration of agonists continues, new neuronal feedback loops may develop at intake locations of the body, either downregulating receptors or inhibiting the sensitivity of those sites [30,73–83]. There are individual variations in the production of binding agents and in the response of OR systems to either an excess or deficiency of opioid binding to ORs, with several alternative options for the adjustment of OR systems. Up- or downregulated OR density therefore might be a candidate factor for individual differences in dispositional emotionality manifesting as temperament traits and emotionality disorders.

*MOPr activation* has, most generally, been linked to positive emotionality, such as feeling pleasure, but also to analgesic effects, relaxation, comfort and affiliative behaviour [7,75–83]. Positive emotionality is, therefore, one of effects of MOPr action but these ORs likely have a more general action described as a calming. Besides, MOPr action not always induces positive emotions: administration of a MOPr agonist in humans reportedly decreased pain but also decreased the participants' mood (i.e. induced negative affect [84]). More consistently studies report that activation of MOPr suppresses HPA arousal, KOPr activation and NA release, which might be mechanisms controlling the stress response, anxiety and flight–fight reactivity, as well as inducing analgesic effects on pain, both physical and emotional [7,73–83]. Evolutionary analysis revealed that the OR systems, including MOPr, are ancient. They emerged in tandem with the immune system, and only later started regulating MA release. Stefano & Kream, for example, pointed out that the ability of MOPr to suppress pain and stress is beneficial in neutralizing an aggressive immune response to injuries and other health challenges, as it decreases pain-related shock which can lead to death [84].

*The action of DOPr* is also associated with pain suppression, with the rewarding and addictive effects of psychostimulants and with antidepressive effects. Since the DOPr system regulates MOPr activation, it is hard to disentangle the effects of these two OR systems [7], but in the absence of MOPr emotional improvement resulting from DOPr action emerges only as a reduction of anxious and depressive symptoms (this is not the same as positive emotionality) [85,86]. Meanwhile the DOPr system expressed more functionality than just emotional regulation as it has been linked to behavioural mobility, and initiation of actions. DOPr agonists stimulated and initiated locomotor activity in swimming and climbing behaviours in rodents [87,88], and high dosages induced convulsions (i.e. uncontrolled motor actions; [85,89]). Mice lacking the DOPr-1 genes showed higher impulsivity, but lower plasticity (a deficient control over when behaviour should start and stop) [85,90]. It is hard to tell to what degree these effects were due to DOPr versus DA action since both DOPr and MOPr regulate DA release [7], which in turn (as noted above) was implicated in behavioural plasticity and prioritizing motor, cognitive, perceptual and motivational elements of behaviour. The association of DOPr action with the initiation of actions rather than emotionality is in line with DOPr being primarily located in the basal ganglia and neocortex (i.e. areas of the brain implicated in the preparation and integration of actions rather than sensory–motivational processes) [91,92]. In fact the thalamus, usually associated with sensory and pain processing, has *the lowest* density of DOPr [92,93], which would not be expected if DOPr action was primarily to control sensitivity to pain or pleasure. However, even for the cases of regions with high density of receptors, see our endnote 1.

Finally, *kappa-opioid receptors (KOPrs)* have been linked to sensory mobilization processes, aversion, chronic anxiety, hallucinations and malaise, activation of NA and stress hormones release and dispositional HPA axis arousal [7,73,74,81,94]. In animals with a deficiency of dynorphin/KOPr action, the expression of stress-related hormones is significantly reduced and in wild animals KOPr antagonists decrease avoidance and anxious dispositions [7,73,74,81,94]. Even though KOPr activation has been consistently linked to chronic anxiety and prolonged stress, this appears to not be the case for acute, situational stress or specific phobias [7,73,74]. Research into the mutual regulation between OR systems and their functionality is still, however, in its early stages and could reveal more complexity than dimensional models can handle. For example, it has been shown that the functional effects arising from the activation of OR systems depend on the degree of OR activation: only high doses of KOPr agonists induce anxiety, while low doses act as an analgesic, and very low doses may induce positive mood states [7,73,95].

To summarize, the following universal functional architecture of human behaviour is proposed—three dynamic functional aspects regulating behaviour (forming the three columns of the FET model, figure 1), taken at several levels of contextual complexity (three rows of figure 1). Emotionality traits (emotional dispositions) are suggested to emerge as amplification of key regulatory tendencies (and linked to dysregulation of OR density): sensory–orientational mobilization (with KOPr leading), subjective comfort and security due to perceived level between needs and capacities (led by MOPr), and selectively acceleration, initiation of actions in tight interplay with DA systems (led by DOPr). FET can be considered as the main structure of psychological taxonomies of healthy people and people with mental illness.

## 2. Troubles with dimensional models

### (a) Dimensionality approach and two main pairs of dimensions

A second, dimensional, approach to psychological taxonomies plots psychological types into dimensional quadrants formed from the extremes of opposite poles along the chosen dimensions. Independence (orthogonality) between parameters (or whatever word we want to use—variables, scales, dimensions, classes, components) is, therefore, important for this approach. The first dimensional model was described by Kant in 1798 [96]. He mapped the four Hippocratic temperament types into the quadrants of two dimensions ‘Energy (Activity)’ and ‘Emotionality’. Two dimensions that are similar to this model (i.e. ‘energetic’ and emotionality-related) have been described in all major models of individual differences (see [17,18] for review). In psychiatry most common models were based on different dimensions related to emotional positive–negative valence. The prototypical models of this second group were offered by Kraepelin and Kretschmer [97,98], and in the 1970–80s temperament models with dimensions of Positive and Negative Affects (PNA) became prominent in psychiatry (for example, [99–104]).

Initially differential psychologists did not take the PNA model seriously since it looked rather simple. After all, Approach–Withdrawal behaviour can be observed even in single cell organisms such as amoebae [105], and intuitively people understood that there is more to biologically-based traits than just emotional valence. Much more credit has been given in the past 20 years to a lexically-derived model, the Five Factor Model of personality (FFM) [106,107]. Personality is, however, a socio-cultural concept,^2^ and the social level of integration of individual differences is affected by pro-social biases, which highlight some traits and downplay others. For example, FFM dimensions resemble societal expectations: sociability, assertiveness, conformity, obedience. Yet, individual differences such as physical endurance, plasticity of actions, and sensitivity to specific reinforcers remain in the shadows. In spite of the rather aggressive spread of the FFM in psychology and its claim for describing ‘human universals’, it is often forgotten that the FFM is primarily a lexical model of peer perception (i.e. of how people see each other and express it in words [106–109], not a model of biologically-based differences. The lexical nature of this model together with people's subjectivity inevitably create several language biases in the resulting factor structure [108–112]. The massive number of cross-cultural studies is often used as an argument for the validation of this model, but these studies are designed to find universal features between cultures, not the structure of individual differences. What are being validated in these studies, therefore, are only the pro-social bias of language, the negativity bias of emotionality (emerging as Extraversion and Neuroticism factors) and people's conflation of traits in their social perception, not taxonomy of biologically-based differences.

Another factor promoting the FFM was the use of factor analysis (FA) and its derivatives, such as structural equation modelling (SEM). These are the most popular mathematical methods in differential psychology (psychology of individual differences), and their use by psychometrists is often confused with the task of classifying individual differences. FA is not used in the mature sciences (biology, cosmology, chemistry, medicine and even mathematics itself) for their classifications owing to the weaknesses of FA: linearity (even so-called nonlinear FA uses linear correlations), an incapacity to deal with highly integrated systems, etc. As described in the Introduction to this theme issue [113], it has been 100 years since the first objections to using FA for psychological taxonomies emerged, and criticism of FA models (such as FFM) has continued [114,115]. When everything is interdependent in biological processes, a correlation-based analysis collapses the complexity of measurements of these systems into a very limited number of dimensions which are not very useful for practical considerations.

The requirement of independence of scales is very convenient for mathematical purposes and is important in psychometric practice except for the fact that it does not really exist in natural systems. For example, the independence of the dimensions in the PNA model appears to be due, not to independence of the underlying regulatory systems, but rather to the fact that these interconnected systems act differently in response to positive or negative events (as discussed below). The priorities of psychometrists and differential psychologists, therefore, differ greatly, and psychometrists or statisticians are the last people to be consulted when developing taxonomies of biologically-based traits. Unfortunately the opposing priorities of psychometricists and differential psychologists are often overlooked: there is a commonly held view in differential psychology that FA results of psychometric studies (i.e. how the items or scales in psychological tests are grouped) provide valid evidence of associations between real neurophysiological processes. This is, perhaps, a reflection of too much public trust being given to self-report tests and verbal descriptors in psychology, and too little awareness of their profound methodological weaknesses in comparison with neurophysiologic experiments. With the development of psychometrics (which demands independence of scales in psychological tests), FA became dominant in differential psychology. These days most of the discussion in this field cycles around the FA structure of self-report tests obtained from various populations (e.g. what facet belongs to what factor as if this facet (or factor) relates to a real neurophysiological system). The confusion of mistaking psychometric evidence for experimental evidence is very wide-spread, ‘trumping’^3^ psychometrically-derived models, such as the recent FFM, to a dominant position in psychology. As Norman & Streiner [116, p. 144] noted, ‘factor analysis … when applied blindly and without regard for its limitations, it is about as useful and informative as tarot cards.’ Indeed, in spite of the ability to group variables in a way that resembles something real, like tarot cards, FA cannot enable us to differentiate between the components of integrated systems. That is what has happened with the two major dimensions of psychological taxonomies, leading basically to a deadlock that appears as a single ‘doing good – doing bad’ dimension.

### (b) Interlocking between two main pairs of dimensions: PNA model is winning

At the end of the twentieth century, several reports showed that the ‘golden pair’ of Kant's ‘Activity + Emotionality’ dimensions might be not independent, belonging instead to one dimension of emotional valence. High performance capacities and approach behaviours were found to be associated with confidence and positive emotionality; emotionality *per se* appeared to have a strong negativity bias [81,117–119]. Longitudinal studies of emotional traits of temperament also showed the consistency of withdrawal, neurotic and shy behaviour throughout the lifespan [120,121]; however there were no reports of a similar consistency for positive emotionality. Studies showed that most neutral verbal materials were perceived more positively by individuals with a high energetic capacity and more negatively by individuals with high neuroticism [49,109,122,123].

These findings led to suggestions that perhaps the two main dimensions of psychological taxonomies should not be Kant's ‘Energy–Emotionality’ dimensions, but instead should be dimensions based on emotional valence. By the end of the twentieth century, a number of Approach/Withdrawal (A/W) models appeared in temperament research, which unified Positive Affect with the Approach/Extraversion (energetic) trait, and Negative Affect with the Withdrawal/Neuroticism (emotionality) trait (for example, [60,99–102,120]). In these models, the ‘Positive Affect/Approach’ dimension included such different characteristics as consistent optimism, approach behaviour, investigative activities (novelty seeking), physical endurance and endurance in social–verbal activities; the dimension of ‘Negative Affect/Withdrawal’ included characteristics of social withdrawal, insecurity, avoidance of uncertainty, low sociability and preferences for individual assignments.

The unification of the PNA with the two main dimensions of the FFM meant that there was no stand-alone dimension describing energetic capacities. This ran counter to solid neurophysiological evidence of the existence of several systems regulating behavioural arousal, anatomic and neurochemical. In addition, it was known that behavioural activation and approach (grouped by these models into Positive Affects dimension) can be accompanied not just by positive but also by negative emotions (such as in aggression). Emotionality, in turn, even with its negativity bias, can often be positive. Gray [124], who also initially proposed a two-dimensional model distinguishing between a Behavioural Activation System (BAS) and a Behavioural Inhibition System (BIS), agreed with Luria [23] that energetic vigilance and HPA arousal are likely two different activation (‘energetic’) systems. He revised his Reinforcement Sensitivity Theory by adding a third component, a Fight/Flight System (FFS), breaking the symmetry of his two-dimensional model.

The unification of the FFM with valence-based models has been adopted by the Research Domain Criteria (RDoC) group working on upgrades of the DSM [103,104]. It was, however, rather disheartening for differential psychologists to see that the outcome of their great labours has been the classification of the complexity of mental disorders by just two valence-based affects. Considering the diversity of brain cells, neurotransmitters, receptors, neural systems and functionality, as well as observable human diversity, we are likely missing something in the current version of the DSM, based on the PNA model.

### (c) Except … there are no specialized systems of Positive/Negative Affects

In spite of providing the main framework for the latest version of the DSM-5, it is embarrassing that the PNA cannot find specific neurophysiological systems that would validate this model. Although many resources have been invested in magnetic resonance imaging (MRI) research, the more results it brings, the more it becomes clear that there are likely no well-defined neuroanatomic systems specialized for Positive and Negative Affects [48–50,125,126]:

(1) *The amygdala likely processes salience and not fear*. Recent studies have shown that activation in the amygdala (AM), which classically is regarded as a structure that processes mainly negative emotions, is associated not only with negative emotionality, but with positive emotionality as well (i.e. optimism [127–129]), following exposure to positive photographs and positive emotional words [51,52,129], anticipation of pleasant tastes [130,131] and positive future outcomes (see [48–50,125,126] for reviews). Moreover, naturally threatening but known situations often do not activate the AM or produce a different pattern in its activation relative to novel objects [48–50,81,125,126]. Several researchers have suggested that the AM processes the novelty or saliency of events, prioritizing incoming information, but not solely emotionally negative aspects of events [47–50,60,81,125,126].
(2) ‘*Reward circuits*’ *are activated in adverse situations as well.* The ventral tegmental area (VTA) and the nucleus accumbens (NAc) are classically regarded as the core of the positive reinforcement loop as they are highly involved in addiction. Activation of dopaminergic neurons in the VTA, however, was found to be similar whether it was induced by either negative experiences (prolonged stress) [132–134], anticipation of aversive stimuli [134] or, as well-known, pleasurable experiences (psychoactive drugs). Some studies report that treatments that increase NAc excitability depressed mood whereas treatments that reduce this excitability appeared to elevate mood [133]. Moreover, ‘reward circuits’ were reported to be activated during an anticipation of getting a reward but not at the receipt of the reward [135], suggesting that they process projection of future events rather than being responsible for inducing specific (positive) emotional valence.
(3) *Hemispheric asymmetry appear to have functionality that is more in line with Kant's dimensions.* In the mid 1980s several researchers linked emotional valence to cortical hemispheric asymmetry. Subsequent analyses, however, showed that the right hemisphere might be dominant not just for negative emotionality, but for emotional processing in general, irrespective of valence; the left hemisphere was linked more to the ability to integrate behaviour rather than to specifically positive affect [136–139]. In the light of these findings it has been proposed that hemispheric lateralization is more closely related to approach versus withdrawal behaviour than to affective valence [136–139].
  Thus, multiple cortical, subcortical and basal ganglia structures appeared to participate in emotionality as multi-stage and multi-layered emotional processes, but at the same time, as Pessoa [125] put it, ‘none of the ‘affective’ structures are purely affective’. Moreover, neurons and nuclear groups that are thought to be specialized for either positive or negative emotionality are often contained within the same gross anatomical structures, including the AM and NAc. More consistently, the functionality of ‘affective’ brain structures has been associated with the assessment of salience and the estimation of probabilities of possible needs and current capacities to meet them.
(4) *Monoamine systems might not be systems of positive or negative emotions either*. Commonly held views attributing Extraversion and Positive Affect to DA system [9,16,19,107,140] were confronted with non-supportive reports [141,142]. Appetitive stimuli appeared to enhance activity in the mesocortical DA system to *a lesser and not higher degree* and for a more transient period than did aversive stimuli [2–4,11,141,142]. Higher DA release was reported not only in positive but also in negative circumstances, such as a defeat, aversive stimuli, stress and foot shock [2,3,11]. There is a growing consensus that the main function of DA release pertains to the attribution of salience and priorities in perception, and to the integration of actions [2–4,8,11,13,15,17,18], which is necessary for behavioural plasticity, integration of behavioural elements, programmes of future actions and motor readiness [2–4,11]. Dysregulation within the DA system was less associated with changes in emotionality and more so with a compromised ability to integrate behaviour adequately to a situation (i.e. impulsivity, rigidity, OCD) [2,3,143]. The remarkable role of DA in ‘salience-labelling’ can be seen in its association with pathological attachment of importance to irrelevant stimuli in schizophrenia [144,145] and psychoticism [146], both linked to an excess of DA but both associated with negative, and not positive, affectivity. Meanwhile some studies of DA D4 receptor genes found no associations with Extraversion [14,15]. Moreover, the early opinion that Extraversion (or general arousal) was based on the cortical-ARAS system was confronted by experiments in neurochemistry that showed that the ARAS system has at least four different sub-systems of arousal, diverging in their functionality and directionality of NT release [11,17].
(5) *OR functionality relates to more than Pleasure–Displeasure and/or Arousal*. The greatest hopes of the Positive–Negative Affect model were associated with OR systems. As described in §1d, the functionality of OR systems extends further than the regulation of emotional valence. At first it seemed that the functionality of KOPr and MOPr systems might be in line with Kant's two dimensions, and this led to the emergence of models mapping emotional states (affects) around a circumplex with the dimensions of Arousal and Pleasure–Displeasure (see [48,147] for reviews). However, it is important to underline that these models relate only to the classifications of affects, and not all temperament differences. For example, KOPr activation was not linked to a general behavioural arousal or a state of wakefulness in a way akin to orexin neurons in lateral hypothalamus and instead was described as primarily sensory mobilization, modified perception and information processing, and chronic anxiety. It has been shown that intravenous administration of KOPr agonists produce not only anxiogenic effects but also perceptual distortion of sensory stimuli, depersonalization, speech processing problems, and thought disorganization (see [94] for review). Such specificity in KOPr action towards the orientational aspects of behaviour might explain why some studies using non-orientational behavioural markers reported no anxiolytic effects due to KOPr [148]. Moreover, the ability of the KOPr system to activate NA release makes it an important player in the amplification of alerting and orienting aspects of behaviour [73,74,94,95]. The MOPr system, as discussed above, was found to have more functionality than just ‘Pleasure–Displeasure’. Owing to mutual KOPr–MOPr suppression [7,79–82], it is not independent (orthogonal) to the KOPr system and acts as a general approver of the current state or of programmes of actions, as a suppressor of pain and stress-related arousal.

Similar to the FET, suggestions that the functionality of endogenous OR systems can relate to more than just emotional valence emerged several decades ago, when differences between MOPr and KOPr actions were associated with differences in the regulation of emotional versus perceptual experience [149]. Moreover, the specific functionality of the DOPr system should be also acknowledged, and therefore two-dimensional models still might be insufficient for the development of psychological taxonomies.

## 3. FC in taxonomies: behaviour is about ‘doing’, and emotionality assists it

### (a) Emotional valence as a multi-systemic estimate of Needs and Capacities (N/C ratio)

**Figure 2.**
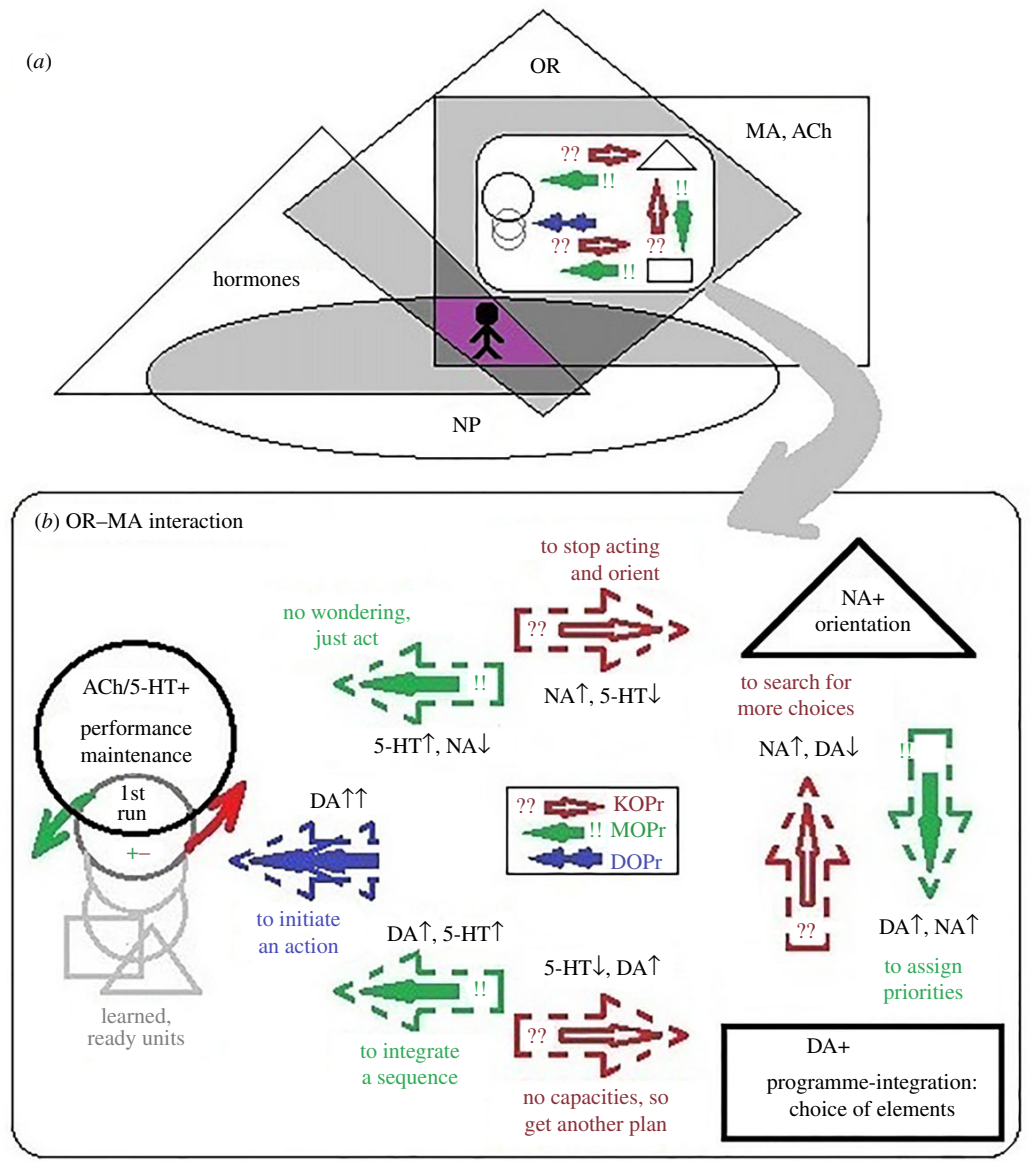
At least four classes of neurochemical systems (hormones, opioid receptors (ORs), monoamines (MAs) and acetylcholine and neuropeptides (NPs)) regulate human behaviour and contribute to consistent individual differences. (*a*) These systems interact with each other; however, they also have their specific functionality. (*b*) As shown in the example of the OR–MA, the directionality of their action follows the pattern of three functional aspects constructing a contingent cycle of action. Normally, (smaller arrows) OR systems regulates MA release without persistent emotional dispositions, in addition to non-OR exchanges between MAs using alternative synaptic mechanisms. The density of ORs might up/downregulate (dashed arrows), inducing dispositional emotionality. 5-HT: serotonin; DA: dopamine; NA: noradrenalin; ACh: acetylcholine; ↓: suppression of release; ↑: activation of release. This pattern is only partial as other mechanisms of transmission are not shown; also there are differences between these processes at the level of cortical versus basal ganglia systems, action of GABA/glutamate mediators and a diversity of receptors within each system. (Online version in colour.)

If there are no specific systems of Positive and Negative Affects, what induces these affects—after all we all experience them, and emotionality should be a part of our psychological taxonomies. There is a consensus among specialists in emotionality research that emotional valence is *generated by multiple processes within the central and autonomic nervous systems*. Not only subcortical limbic and basal ganglia but also cortical structures were found to contribute to it, making it a derivative of subjective estimation of capacities and situational needs [48–50,60,81,125,126]. Neuroanatomic and also multiple neurochemical systems have been linked to emotional valence: monoamines, hormones, GABA/glutamate, neuropeptides including ORs, BDNF and CREB (figure 2*a* relates to four of these systems). None of these systems works independently in generating either positive or negative emotionality; instead, each of these systems casts its vote in the final emotional summary, and such ‘voting’ comes with functional specificity (sensory, motor, cognitive) related to the systems in which given receptor groups are located.

In terms of Negative Affect, this is likely the first general reflection when there is something wrong with any of the neurophysiological systems of behavioural regulation. Buddhist philosophy was perhaps the first to emphasize that happiness or suffering (i.e. emotional valence) is a contingent interplay between at least two systems: (i) desire, or strength of a need (*N*), and (ii) estimation of the capacity (*C*) to meet this need. In psychophysiology a similar idea emerged in the mid 20th century in the experimental work of psychophysiologists Anokhin [22] and Simonov [60], and that of multiple brilliant authors who also considered emotional valence to be a capacities-based estimator of future success or failure [48–50,150–152]. With *C* > *N* the resulting valence is positive, and with *C* < *N* the valence is negative. This formalism seems to be in line with the described relationship between the emotionality and energetic temperament traits: high capacities signal control over the events, low capacities signal lack of control. It also explains the negativity bias of emotionality, especially in cases of novelty: novelty means un-preparedness to handle the situation, even a pleasant one, and only when people recognize their own capacity to handle it might the initial negative reaction subside. An inability to handle negative events might have more serious consequences for an animal's survival than an inability to handle positive events, and this might explain the negativity of the default emotional reaction to novelty [81,126]. The Affect-based dimensions are, therefore, too general and not very informative as they are the product of the action of multiple neurophysiological systems.

In the FET, it is suggested that KOPr action in bringing the *N*/*C* ratio to balance is, in the *C* < *N* case, to increase *C* by alerting and orienting the nervous system to additional alternatives, and in the *C* > *N* case, to increase challenges that would employ existing capacities. A dysregulation in the density of KOPr and adrenergic receptors might lead, therefore, to emotional dispositions of Neuroticism and Sensation Seeking. Neuroticism is a traditional temperament trait that was also noticed in the FFM, so it is not surprising that it was proven to be biologically based and consistent over a person's lifespan in longitudinal studies since the mid 1960s [120]. Negative Affect cannot be equated with Neuroticism because dysregulation in virtually any neurochemical system could bring about negative emotions or Negative Affect, signalling to the person that ‘something is not right’. In contrast, Neuroticism is more specific, referring to the avoidance of novelty and uncertainty and to enhanced perceptual alertness.

### (b) OR-MA interaction follows the logic of functional constructivism

The pattern of OR–MA actions appears to be in line with the functional roles of the MA. Figure 2*b* considers the example of OR–MA interaction and its relationship to the functional blocks of action construction as described in the current literature. Smaller arrows inside the dashed arrows between blocks symbolize the normal regulation of MA release by the ORs, and enlarged dashed arrows symbolize cases of dispositional emotionality. The FET summarizes evidence showing that OR systems control the MA releases, which, in turn, regulate functional aspects of behaviour (indicated in the figure as examples). of behaviour (indicated in the Figure as examples). In brief, MOPr and DOPr induce the release of DA but specificity between regions has also been reported [7,30,79,153]; MOPrs also (selectively and indirectly) modulate 5-HT release but inhibit NA release [7,75,80–84,154]; KOPrs act in the opposite direction, inducing NA release, suppressing DA release and acting selectively on 5-HT [7,73–83,91,94,153,154]. As illustrated in figure 2*b*, ORs act on the three MA systems in a very specific way: KOPr action on the three MA can be summarized as being directed to the expansion of behavioural alternatives (‘change if you cannot accept it’); DOPr action initiates the behaviour and MOPr activation works as the approval of current choices (‘accept if you cannot change it’). Capacities of MA (as well as neuropeptides and hormones) systems determine what part of this contingency cycle the behaviour (depicted by seven possible regulatory aspects) will be running smoothly and where it will be stuck, and the action of ORs is directed along the same functional aspects.

The pattern shown in figure 2*b* shows how the OR system (capable of inducing dysphoria, pleasure or agitation) and the MA system (regulating formal aspects of activities) are intertwined. However, as depicted in figure 2*a*, several systems functionally overlap in emotional regulation but each of these systems has multiple functions outside of the regulation of emotions and so *their functional overlap in emotionality is only partial*. In terms of OR–MA regulation, in addition to OR action, MA systems use alternative synaptic mechanisms (such as ionotropic ligand-gated receptors, additional neurotransmitter systems (GABA and glutamate) or non-OR GPCR systems) for their MA-to-MA connections. GABA(A) receptors were found to have significant co-localization with MOPr and KOPr receptors as well as MA [7,75]. Moreover, the OR–MA regulation often involves major players in the immune and metabolic systems, and in this case the OR action on the MA is really represented by the capacities of the body to produce needed behaviour (i.e. ‘body bias’). This makes MA and OR systems partially coupled (which is seen in evaluative biases in cognition [50,125]) yet still partially independent in the regulation of behaviour. Therefore, *we cannot see them as specialized neurophysiological systems of PNA*. For example, MOPr regulates DA release, and this might be a reason why DA release is often associated with positive emotionality. However, DA and MOPr do not always work together or in unison. Decoupling of the action of DA and MOPr was demonstrated in Berridge's study [155] in which the mesolimbic DA system was removed in rats. These rats had normal *liking* reactions to sweetness but their *wanting* (planning and prioritizing) behaviour was compromised. An almost reversed situation, with an intact DA system being electrically stimulated, caused rats to eat more (i.e. to initiate actions related to stimuli) but did not improve their liking. In turn, when MOPr action was suppressed by using KOPr activation (since KOPr and MOPr suppress one another using direct and indirect mechanisms), DA initiation of behaviour according to previously integrated programmes was not affected [94]. This demonstrates that even though MOPr controls DA release, resulting in the association of goal-setting processes with positive emotions, these two systems have different functionality: DA prioritizes and sequences actions, while MOPr ‘sweetens the deal’, approving the behavioural priorities.

When it comes to Positive Affect, the name ‘reward networks’ for DA systems in the NAc, ventral pallidum and VTA appears misleading (originating in addiction research). Indeed, activation within the these networks during exposure to desirable objects—specific food, addictive drugs, sex—is often interpreted as evidence for this network being the Positive Affect system. However, this system is likely just part of a global system of behavioural integration, and as a matter of course is activated during exposure to an individual's priorities, which should be part of their programme of actions. In fact, damage to the NAc or VTA can produce a state of profound torpor, not a loss of pleasure. In Parkinson's disease, which affects DA projections in the basal ganglia, it is the ability to prioritize but not to execute motor actions that is compromised (named about 100 years ago as a ‘paralysis of the will’ [156]). The basal ganglia are known for control of both motor and motivational features of habits, learned units of behaviour that cortical systems use as building blocks for the integration of complex behavioural programmes. Execution of habitual behaviour proceeds faster than behaviour that requires orientation because habits are regulated by striatal systems having less cortical control than those for highly contextual behaviour. This is likely a factor in habit-related mental disorders, such as OCD or addictions. Both OCD patients and drug addicts (who have dysregulated functioning of dopaminergic ‘reward loops’) commonly report a lack of pleasure in executing their obsessions but at the same time an inability to stop their habits and a feeling of being trapped. MOPr and DOPr are located mostly in executive areas of the human brain (such as the frontal lobes and basal ganglia) [91,92], suggesting that their main function is not to induce pleasure *per se*.

As figure 2*b* illustrates, MOPr action likely generates a state of approval of behavioural alternatives; DOPr action initiates chosen alternatives, and this might explain the coupling between positive mood and planning or feeling sufficient capacities to satisfy the needs. The ‘approval’ function of MOPr is observed even in the case when chosen actions are very minimal. For example downregulated MOPr density (due to chronic administration of endorphins) in marijuana smokers induces a chronic ‘approval’ of their behavioural choices often consisting of watching TV all day long, living on government benefits and continuing ‘planning’. In cases of extreme incapacities, such as physical or emotional pain, MOPr action is analgesic, sending a deceiving but beneficial impression to the individual [84].

### (c) Evaluative bias in human perception likely affects lexical psychological taxonomies

When we derive the dimensionality of regulatory systems from human observations (the subjectivity of which affects the choice of variables in FA) there is a real danger of being influenced by the emotionality biases inherent in human cognition [109–113]. Since the work of Kelly [157], it has been known that humans tend to employ bipolar scales (positive–negative criteria) in all of their judgements first, and only later do they use more practical, non-evaluative criteria. In the dimensionality approach, both models—the FFM and PNA—use descriptors derived from human judgements, which universally suffer from evaluative bipolarity. When FA is applied to such data, the evaluative biases of descriptors will guarantee the creation of valence-based categories missing non-evaluative features of the real structure of the systems that humans are evaluating. This evaluative bias creates the first impression that OR–MA teams (for example, KOPr–NA or DOPr/MOPr–DA) are neurophysiological correlates of Negative and Positive Affects, or Neuroticism and Extraversion, and overshadows the fact that there are at least three, and not two OR–MA teams, and that functionality of either OR or MA extends beyond emotionality or socialization (i.e. includes orientation, initiation, prioritization, analgesic effects, etc.).

For example, Impulsivity was long noted to be a part of Extraversion [106,107] but it is often associated with Negative, and not Positive Affect. As another example, the Sensation Seeking trait initially was a facet of the factor of Extraversion (unified with the Positive Affect dimension) whereas high Sensation Seeking is associated with complaints of boredom and a need for fast ‘mood-fixers’ (i.e. with Negative Affect). In fact, studies using subjects with low endorphins showed that they had high Sensation Seeking and dysphoria but low Neuroticism [158,159], and therefore Sensation Seeking cannot be classified as being a facet of either Positive Affect or Neuroticism (as a part of Negative Affect). As a third example, high sociability is also a trait of Extraversion and Positive Affect whereas in reality there is the phenomenon of ‘misery likes company’ (i.e. negative affectivity motivating people to pursue socialization and social approval). The dimensions of Positive/Negative Affects, therefore, appears to be too general and not be very useful for psychological and psychopathological taxonomies.

### (d) Dysregulation in receptor density: from temperament traits to mental illness

In applying the FC perspective, we suggest that, whether in a positive, negative, or emotionally neutral state in processing current events, the psyche tries to answer the question ‘what should I *do with it*?’, and not ‘how does this makes me *feel*?’ The main priority of the nervous system is to assist in the construction of behaviour, and features of biologically-based traits were developed in evolution around this main priority. Emotionality developed in evolution much later than physical and cognitive regulation of behavioural construction and therefore we should not base the main dimensions of psychological taxonomies on emotional valence.

FC considers behaviour as the constant production of neurophysiological contingency cycles based on the capacities of neurochemical factories having their own recovery processes, outlier states, compensatory mechanisms for various inevitable temporary deficiencies, and tight interactions between their components. When something is consistently wrong with these compensatory mechanisms, OR and other GPCR systems up- or downregulate while trying to restore MA release to its balanced cycles (depicted as enlarged dashed arrows in figure 2*b*). Dysregulation in OR density can induce dispositions for chronic sensory arousal (KOPr system), impulsivity (DOPr) or relaxed attitudes (MOPr), and dysregulation in MA receptor density can induce consistent non-emotional dispositions, examples of which are given as text labels near these arrows. Slight dysregulation in MA receptor density can emerge as non-emotionality traits and changes in OR systems—as emotionality-related temperament traits listed in figure 1. Extreme cases of dysregulation in receptor density have been linked to mental illness, such as Borderline Personality Disorder and attachment disorders (MOPr system [76–78,81,160], chronic anxiety (KOPr system, [7,73,81,94]) and impulsivity (DOPr system, [87,90]). A majority of symptoms of mental illnesses as listed in the DSM/ICD could be classified as belonging to at least one FET component. There is, therefore, a potential in using the FET framework for classifying mental disorders, with several studies having been carried out based on this perspective (see William Sulis's contribution to this issue [161]).

As applied to the general population, however, there is a ‘grey area’ between mental illness and healthy temperaments, with many shades of grey in which the ‘chemical factories’ of people do a ‘good enough’ or barely ‘good enough’ job in dealing with situational changes. To deal with such changes our ‘chemical factories’ have several contingencies, and the way people respond to contingencies should be included in the descriptors of their individuality. Those days in which ‘something comes up’ are, unfortunately, more common than exceptional, and, therefore, the concept of ‘traits’ as the most frequent behavioural patterns should cover a wider range of contingencies of human functioning than is usually implied. Instead of being overwhelmed by the diversity of specific situations, the FET model suggests focusing on the formal dynamic features of situational demands, making them compatible with features of temperament (i.e. capacities of an individual to handle these demands). In this way, both individual differences in behaviour (abilities) and the context/properties of the tasks (demands for abilities) could be represented in the FET matrix in terms of compatible components reflecting contextual complexity, uncertainty, dynamics, duration, physical-versus-social-versus-mental aspects of behaviour, and degree of urgency. Echoing Luria's [23] term ‘working mosaic of the brain’, the FET model suggests that its components work in neurochemical ensembles, simultaneously regulating the construction of actions in their specific ways.

## 4. Conclusion

Taxonomies of psychological individual differences should take into consideration the main principle organizing neurophysiological regulatory systems: that they all are shaped to facilitate the generation and integration of behaviour. From the functional constructivism perspective it appears that the universal architecture of the action construction could provide the main framework for taxonomies of healthy individual differences and mental illnesses. This paper has reviewed the entanglement between Affect-related dimensions and dimensions related to energetic capacities, and linked this entanglement to the interaction between the OR and MA systems. As discussed, emotional valence is likely a first estimate of needs and capacities for handling these needs by an individual. The OR–MA interaction contributes just a part to this estimate which actually involves multiple neuroanatomic and neurochemical systems. There is much more to behavioural regulation than emotionality, and both OR and MA systems have been linked to numerous functional and non-emotionality aspects of behavioural regulation. There are no specialized systems of Positive or Negative Affect. Emotional estimates assist cognition in forming a first draft of an action plan, but neither emotionality nor cognition substitutes for the construction of behaviour. In most healthy persons and even in some mental disorders this construction is accompanied by neutral emotionality. Thus both psychological taxonomies and PNA-based classifications of mental disorders should be revised in tune with the non-emotional, functional aspects of behaviour.

The interlocking between emotionality and executive capacities, as well as interactions between orientation, integration and the energetic maintenance of behaviour, should be taken seriously in our taxonomies. For us to progress scientifically, it pays to keep track of how functional, non-emotional aspects of behaviour contribute to the resulting emotional valence, and vice versa (i.e. how dysregulation in OR density creates emotional dispositions that affect non-emotional aspects of behaviour).

Owing to the high interconnectivity between regulatory systems, correlational analysis and its derivatives (FA or SEM) are rather useless for deriving taxonomies. Close inter-relationships between the functional features of behaviour will always result in two clusters of characteristics, not far removed from the ‘good–bad’ categories in the perception of early humans. At the present time differential psychology indeed seems to be imprisoned by the FA-based dimensionality approach, which is blind to the functional relationships between the components of human individuality. It confuses them with lexically-derived personality models of social perception, such as the FFM. Statistical methods cannot provide our classifications as they depend on us to sort out the variables before we apply these methods, and these variables are exactly what we are missing in our taxonomies. This means that we still have a lot of work to do, with a long walk ahead along the road of functionality of neurophysiological systems.

## Supplementary Material

Figure 1 A and B in a form of a large Table.

## Endnotes

1 Whenever the functionality of NT systems is discussed here, keep in mind that there are several stages in the release of each of these NTs, which involves a cascade of variable and optional transformations between GABA/glutamate, enzymes and metabolites, GPCRs, BDNF, CREB, calcium and other chemical systems, including partner monoamines. Moreover, the diversity of receptors within the same systems, and their different actions in different brain structures, create another serious challenge for understanding the functionality of MA systems.

2 The FFM supporters defend their consistent omitting of the concept of temperament on the basis that personality is a product of integration between temperament (or other biological factors) and socio-cultural factors, and therefore there is no possibility to separate these factors. However, sex, age and mental illness (also based on neurochemical and physiological systems) interact with socio-cultural factors as well so they also contribute to personality but we do not blend these concepts. There are benefits, therefore, to keeping our concepts differentiated. Temperament, in line with its original concept, is defined here as neurochemically-based individual differences noted in both pre-cultural individuals (animals, infants) and adult humans, whereas personality is a socio-cultural concept describing individual differences primarily in humans. Temperament traits include consistent *formal-dynamical* individual differences in behavioural regulation (endurance, speed of behavioural integration, reactivity, sensitivity to specific reinforcers, emotionality), whereas personality classically is considered as comprising the contents characteristic of human behaviour (such as values, attitudes, habits, preferences, personal history, self-image, etc.) [18].

3 We can use the term ‘trumping’ in the sociology of science for cases of aggressive spreading of rather weak theories owing to their media advantage while stronger theories are pushed into the shadow. A trump card in card games refers to a card of low value that can take a leading position, overpowering more deserving cards merely because of a temporary advantage. In our time, when educational systems around the world suffer from insufficient funding of biological studies and evolutionary research, it creates an advantage for relying upon easy-to-use statistical methods and simplified models, rather than experimental research.

## Data accessibility

This article has no additional data.
