# Supplementary material for: Functionality versus dimensionality in psychological taxonomies, and a puzzle of emotional valence
Source: Philos Trans R Soc Lond B Biol Sci. 2018 Feb 26;373(1744):20170167. doi: 10.1098/rstb.2017.0167 (PMC5832691; doi:10.1098/rstb.2017.0167)
Supplement: Figure 1 A and B in a form of a large Table. [file rstb20170167supp1.doc]

**Supplementary material.**

Table S1A. Comparison of neurophysiological and temperament models in a framework of components of the Functional Ensemble of Temperament (FET) related to physical-motor aspects of behavior.

| ***Functional aspects*** | ***Behav. orientation*** | ***Speed of intergration*** | ***Maintenance*** |
| --- | --- | --- | --- |
| *Traits in FET model:* | *Sensation Seeking* | *Motor-physical Tempo* | *Motor-physical Endurance* |
| Leading neuro-transmitters: | NA, AdrR1 | DA, PRL | NP, 5-HT, Ach |
| Was the trait observed in animals /young children? | Zuckerman, 94:  Animals, children | Sports, breeding practice  Animals, children | Pavlov 41, sports  Animals, children |
| **Neuro** | **psychological** | **models:** |  |
| Pavlov,1906-1935 |  | Mobility | Strength of nerv. system |
| Anokhin, 1935 | Afferent synthesis |  | Execution block |
| Luria, 1948-70 | Information-sensory block |  | ARAS→cortex: Energetic block |
| Teplov,1947-61 |  | Mobility of varios types | Strength of excitation |
| Nebylitsyn, 1963 |  | Lability | Strength of excit |
| Gray, 1982 | BAS |  | BIS |
| Cloninger, 1987, 94 | Novelty Seeking /DA |  |  |
| Rusalov, 1989, 1997 |  | Motor tempo | Motor ergonicity |
| Netter, 1991 |  | DA, 5HT, ACh | NA, DA |
| Posner, 1995 | Orienting network |  | Executive net-rk |
| Halgren & Marinkovic 1995 | Orienting system | Response choice system | Sustained behavior sys |
| Robbins & Everitt, 96 | NA systems | DA systems |  |
| Jacobs & Azmitia, 92 | NA systems |  | 5-HT systems |
|  | **Develomental** | **psychology** | **models** |
| Chess & Thomas 1996 | Distractibility | Rhythmicity | Activity |
| Buss, Plomin, 19 84 |  |  | Activity |
| Rothbart, 1988 | Orienting sensitivity |  | Activity, arousal |
|  | **Differential** | **psychology** | **models** |
| Stern 1900 | Sense receptivity | Reaction | Psychic energy |
| Wundt,1902 |  |  | Excitability |
| Heymans,1910 |  |  | Activity |
| Lazursky 1921 | Sensitivity | Speed of actions | Intensity of activity |
| Jung 1923 | Sensing |  |  |
| Kretschmer 25 |  | Psychic tempo | Cyclothymia |
| Adler 1925 |  |  | Energy |
| Cattell 1965 |  |  | Vigilance |
| Thayer 1978 |  |  | Energetic arousal |
| Strelau 1983 |  |  | Strength of excit-n |
| S. Eysenck, 85 | Venturesomness |  |  |
| Tellegen 1985 |  |  | Drive |
| Big Five 1949-1993 | Openness to Experience |  |  |
| Hough 1992 |  |  | Potency |

Note: *- an opposite pole of the trait is compared here to a similar FET trait; 5-HT: serotonin; DA: dopamine; NA: noradrenalin, ACh: acetylcholine; NP: neuropeptides inluding orexins; PRL: prolactin; AdrR: adrenergic receptors

Table S1B. Comparison of neurophysiological and temperament models in a framework of components of the Functional Ensemble of Temperament (FET) related to social-verbal aspects of behaviour

| ***Functional aspects*** | ***Behav. orientation*** | ***Speed of intergration*** | ***Maintenance*** |
| --- | --- | --- | --- |
| *Traits in FET model* | *Empathy* | *Social-verbal Tempo* | *Social-verbal Endurance* |
| FET hypothesis on leading neuro-transmitter systems | NA, OXY | DA, PRL | 5-HT, OXY, VPS |
| Was the trait observed in animals/young children? | Rizzolatti et al 1999  Animals, children | Kuhl et al 2005  Children | Buss, Plomin, 1984: Children |
|  | **Developmental** | **psychology models** |  |
| Kagan, 1962,19 94 | Dependency |  |  |
| Buss,Plomin, 1984 |  |  | Sociability |
| Rothbart, 1988 | Orienting sensitivity |  | Affiliativeness |
|  | **Differential** | **psychology models** |  |
| Stern, 1900 |  | Psychic tempo | Psychic energy |
| Heymans,1910 | Feeling |  | Activity/Drive |
| Spränger, 1914 | Religious | Social | Social |
| Jung, 1923 | Extraversion |  |  |
| Kretschmer, 1925 |  | Psychic tempo | Cyclothymia |
| Adler, 1925 | Social feeling |  |  |
| Cattell, 1965 | Rule Con-scientious | Social boldness | Liveliness |
| Eysenck,1967 | Extraversion | Extraversion | Extraversion |
| S. Eysenck,19 85 | Empathy |  |  |
| Cloninger, 1987 | Reward dependence |  |  |
| Rusalov, 1989 |  | Social tempo | Social ergonicity |
| Big Five, 1949-1993 | Agreeableness | Extraversion | Extraversion |
| Hough, 1992 | Agreeableness |  | Affiliation |
| Taylor & Morrison 1992 | Sympathy | Expressiveness | Social activity |
| Cloninger, 1994 | Cooperativeness |  |  |
| Eysenck, 1995 (EPP) | Non-conformity* | Expressiveness, Manipulativeness | Sociability |
| Akiskal, 1998 |  |  | Cyclothymia |
| Depue & Collins, 1999; Bielsky & Young, 2004 | Affliation (OXY, MOP) | Extraversion (DA) | Affliation (DA, OXY) |
| Baron-Cohen, 2003 | Empathising |  |  |
| Zuckerman, 2002 |  |  | Sociability |

* - an opposite pole of the trait is compared here to a similar FET trait; 5-HT: serotonin; DA: dopamine; NA: noradrenalin, PRL: prolactin; OXY: oxytocin.

Table S1C. Comparison of neurophysiological and temperament models in a framework of components of the Functional Ensemble of Temperament (FET) related to mental aspects of behaviour requiring higher cortical activity

| ***Functional aspects*** | ***Behav. Orientation*** | ***Intergration*** | ***Maintenance*** |
| --- | --- | --- | --- |
| *Traits in FET model* | *Sensitivity to Probabilities* | *Plasticity, re-programming* | *Attention/mental endurance* |
| FET hypothesis on leading neuro-transmitter systems | NA, DA, 5-HT, ACh | DA, 5-HT | ACh, NA |
| Was the trait observed in animals/young children? | Gopnik et al 2001:  Infants | Pavlov, 41, etc; sports. Animals, children | Robarts et al 2000  Children |
|  | **Neuro-** | **psychological** | **models** |
| Luria, 1948-70 | Information-sensory block | Integration-programming block |  |
| Rusalov, 1989 2007 |  | Plasticity in 3 areas | Intellectual ergonicity |
| Netter, 1991 |  | DA, GABA | NA, DA |
| Posner, 1995 | Alerting networks | Orienting networks | Executive networks |
| Halgren & Marinkovic,95 | Orienting system | Event integration system | Sustained behavior system |
| Robbins & Everitt, 1996 | NA, DA | DA | Ach, NA- |
|  | **Developmental** | **psychology models** |  |
| Chess & Thomas, 1996 |  | Adaptability | Persistence/  Attention |
| Rothbart, 1988 | Orienting sensitivity |  | Effortful control |
|  | **Differential** | **psychology** | **models** |
| Stern, 1900 | Association | Combinatorial ability | Attention |
| Heymans,1910 | Reasoning |  |  |
| Spränger, 1914 | Theoretical |  | Economic |
| Lazursky, 1921 | Combinator.ability | Will-power | Attention |
| Jung, 1923 | Introversion |  | Thinking |
| Kretschmer, 1925 | Schizothymic | Psychomotility |  |
| Adler, 1925 | Functional finalism | Creative Self |  |
| Cattell, 1965 | Apprehension | Openness to Change | Perfectionism |
| Strelau, 1983 |  | Mobility | Strengh of inhibition |
| Big Five, 1949-93 | Conscientiousness |  |  |
| Hough, 1992 | Dependability* | Intellectance | Locus of control |
| Taylor & Morrison, 1992 | Objectivity |  |  |
| Strelau & Zawadzki,1993 |  | Perseverance* |  |
| Zuckerman 1994 | Experience seeking |  |  |
| Cloninger 1994 |  | Self-Directedness |  |
| Baron-Cohen 2003 | Systemizing |  |  |
| Eysenck,1995 (EPP) | Irresponsibility*; Practicality | Dogmatism*, Manipulativeness | Obsessiveness |

* - an opposite pole of the trait is compared here to a similar FET trait; 5-HT: serotonin; DA: dopamine; NA: noradrenalin; Ach: acetylcholine

**Table S1D.** Comparison of neurophysiological and temperament models in a framework of components of the Functional Ensemble of Temperament (FET) related to emotionality aspects of behavior

| ***Functional aspects:*** | ***Emotional amplifiers of…*** | | |
| --- | --- | --- | --- |
|  | ***orientational need*** | ***speed of intergration*** | ***capacities/ control*** |
| *Traits in FET model* | *Neuroticism* | *Impulsivity* | *Self- confidence* |
| Leading NT systems: | KOP↑, 5-HT↓ | DOP↑, 5-HT↓ | MOP↓, 5-HT↓ |
| Ttrait observed in animals/infants? | Kagan 1994  Children | Neurophysiology:  Animals, children | Thomas & Chess, 1977: Children |
|  | **Neuro-** | **physiology** | **models:** |
| Pavlov,1906-35 |  | Balance |  |
| Anokhin,1935 |  |  | Feedback block |
| Luria, 1948-70 |  | Energetic block-2 | Limbic → ARAS, |
| Gray 1982 | Behavioral Inhibit. Sys | Behavioral Activ. Sys | Behavior. Activ. Sys |
| Rusalov, 1989, 1997 | Emotionality, 3 types |  |  |
| Netter, 1991 | E↑, NE↓ | 5-HT ↓ | E↓, NE↑ |
| Russell & Barrett, 1999 | Pleasure-Displeas. | Arousal | Pleasure-Displeas. |
| Depue et al, 1999 2005 |  | Constraint (5-HT↓)* |  |
|  | **Differential** | **psychology** | **models** |
| Stern, 1900 | Feelings; sense receptivity |  |  |
| Wundt,1902 |  | Lability of emotions |  |
| Heymans, 1910 |  | Emotional stability |  |
| Spränger, 1914 | Aestetic? |  | Political |
| Lazursky, 1921 | Affect arousability | Impulsivity | Volume of interest |
| Kretschmer, 1925 |  |  | Psychaesthesia * |
| Adler, 1925 | Inferiority complex | Aggression drive | Striving for superiority |
| Thurstone, 1953 |  | Impulsive | Dominant |
| Guilford & Zimmerman,56 | Nervousness, inferiority | Emotional stability | Masculinity |
| Cattell, 1965 | Tension | Emotional stability | Dominance; selfreliance |
| Eysenck,1967 | Neuroticism | Extraversion |  |
| Thayer, 1978 |  |  | Tense arousal |
| Zuckerman, 1979 |  | Disinhibi-tion |  |
| Eysenck & Eysenck,1985 |  | Neuroticism | Psychoticism |
| S. Eysenck, 1985 |  | Impulsiveness |  |
| Tellegen, 1985 | Negative emotionality | Constraint | Positive emotionality |
| Cloninger, 1987 | Harm Avoidance |  |  |
| Strelau & Zawadzki, 93 |  | Emotional reactivity |  |
| Big Five, 1949-1993 |  | Neuroticism | Extraversion |
| Hough, 1992 | Adjustment | Individualism | Achievement |
| Taylor & Morrison, 92 | Nervousness | Hostility, Impulsivity | Domi-nance |
| Cattell, 1965 | Tension | Emotional stability | Dominance, SelfReliance |
| Eysenck,1967 | Neuroticism | Extraversion |  |
| Thayer, 1978 |  |  | Tense arousal |
| Cloninger, 1993 | Harm avoidance | Reward dependence | Self-direc-tedness |
| Carver& White; 1994 | Harm avoidance |  | Reward responsiveness |
| Eysenck,1995 (EPP) | Anxiety; guilt; unhapp-ss | Impulsivity; aggres-n | Ambit-n; assert-ss; infer-ty* |
| Mehrabian, 1996 | Displeasure |  | Dominance |
| Akiskal, 1998 | Anxiety | Irritability | Hyperthym. |
| Clark & Watson, 1999 | Negative affect | Disinhib.-constraint | Positive affect |
| Zuckerman, 2002 | Neuroticism-Anxiety | Impulsivity-SS | Aggression-Hostility |
|  | **Developmental** | **psychology** | **models:** |
| Thomas & Chess, 1977 | Withdrawal | Mood Quality | Approach |
| Buss & Plomin, 1984 | Emotionality | Impulsivity | Anger |
| Kagan, 1962, 19 94 | Anxiety | Aggression |  |
| Derryberry & Rothbart, 88 | Negative Affect | Effortful control * | Extraversion/ |

* - opposite pole of the trait; 5-HT: serotonin; DA: dopamine; NE: norepinephrine, E: epinephrine; Glu: glutamate, NP: neuropeptides, MOP, KOP, DOP: mu-, kappa and delta-opioid receptors correspondingly; ↓: downregulation, ↑: upregulation of OR density.

(Adopted from: Trofimova I. 2016 The interlocking between functional aspects of activities and a neurochemical model of adult temperament. In: Arnold MC (Ed.) *Temperaments: Individual Differences, Social and Environmental Influences and Impact on Quality of Life*. Nova Science Publishers, Inc., NY, USA)
